# Author’s reply

**Published:** 2011

**Authors:** Nataraj Madagondapalli Srinivasan, Akshay Kumar

**Affiliations:** Department of Anaesthesiology, Kasturba Medical College, Manipal, India

Dear Editor,

Appreciating the author’s interest[1] in the case report,[2] we feel that their recommendation of observing the pulsatile column of fluid in the infusion set is quite subjective when the blood pressure is low and fluid is not freely flowing, compared to a chest radiograph. Also, the tip of the catheter might be abutting the wall when the column does not pulsate.

Comparison of arterial blood gases obtained from the catheter to that from the periphery artery might be feasible when access to the site is easy. In a hemodynamically unstable patient, it might not be possible always.
